# Pricing through health apps generated data—Digital dividend as a game changer: Discrete choice experiment

**DOI:** 10.1371/journal.pone.0254786

**Published:** 2021-07-26

**Authors:** Alexandra Heidel, Christian Hagist, Christian Schlereth

**Affiliations:** 1 WHU –Otto Beisheim School of Management, Chair of Economic and Social Policy, Vallendar, GER; 2 WHU –Otto Beisheim School of Management, Chair of Digital Marketing, Vallendar, GER; Universitat Jaume I, SPAIN

## Abstract

**Objectives:**

The objective of this paper is to study under which circumstances wearable and health app users would accept a compensation payment, namely a digital dividend, to share their self-tracked health data.

**Methods:**

We conducted a discrete choice experiment alternative, a separated adaptive dual response. We chose this approach to reduce extreme response behavior, considering the emotionally-charged topic of health data sales, and to measure willingness to accept. Previous experiments in lab settings led to demands for high monetary compensation. After a first online survey and two pre-studies, we validated four attributes for the final online study: monthly bonus payment, stakeholder handling the data (e.g., health insurer, pharmaceutical or medical device companies, universities), type of data, and data sales to third parties. We used a random utility framework to evaluate individual choice preferences. To test the expected prices of the main study for robustness, we assigned respondents randomly to one of two identical questionnaires with varying price ranges.

**Results:**

Over a period of three weeks, 842 respondents participated in the main survey, and 272 respondents participated in the second survey. The participants considered transparency about data processing and no further data sales to third parties as very important to the decision to share data with different stakeholders, as well as adequate monetary compensation. Price expectations resulting from the experiment were high; pharmaceutical and medical device companies would have to pay an average digital dividend of 237.30€/month for patient generated health data of all types. We also observed an anchor effect, which means that people formed price expectations during the process and not ex ante. We found a bimodal distribution between relatively low price expectations and relatively high price expectations, which shows that personal data selling is a divisive societal issue. However, the results indicate that a digital dividend could be an accepted economic incentive system to gather large-scale, self-tracked data for research and development purposes. After the COVID-19 crisis, price expectations might change due to public sensitization to the need for big data research on patient generated health data.

**Conclusion:**

A continuing success of existing data donation models is highly unlikely. The health care sector needs to develop transparency and trust in data processing. An adequate digital dividend could be an effective long-term measure to convince a diverse and large group of people to share high-quality, continuous data for research purposes.

## Introduction

The COVID-19 crisis highlighted the importance of using the potential of patient generated health data (PGHD) not only to track personal encounters, but also for big data research and to understand various under-researched conditions that lead to acute or chronic diseases. For example, the Robert Koch Institute (RKI) has launched a COVID-19 data donation app in Germany [1]. Users may allow the app access to their wearables and donate any data recorded from health apps or tracking devices. The use of health apps and wearables enjoys ever-increasing popularity within society [2]. However, the willingness to donate self-tracked data might be of short duration, considering the special circumstances of the COVID-19 pandemic and the therefore current high level of trust the RKI holds within German society. A more sustainable and lasting solution to gain access to self-tracked data for research and development (R&D) purposes might be the payment of monetary compensation—a digital dividend.

The lack of transparency concerning data processing among some health app providers triggers trust issues and data privacy activism in most countries that are members of the Organization for Economic Co-operation and Development (OECD). Calls for compensation strategies such as a digital dividend are increasing steadily, as evidenced in German political debates about regulatory possibilities of such a dividend [3]. In most OECD countries, personal data is understood to be personal property. Regulations, such as the European General Data Protection Regulation (GDPR) and its translation into German national law, DSGVO, have evolved to protect the individual’s data property, which means that a digital dividend becomes an ever more realistic future scenario.

Health data is understood to be among the most valuable personal data. The pharmaceutical industry in particular could experience significant benefits from engagement in big data research of realistic daily health data and increased efficiency over long and costly clinical trials as a result [4]. German government aims to become a data hub and buy large amounts of personal data as part of their digital strategy [5]. In December 2019, Germany passed the digital care act (Digitale Versorgung Gesetz, DVG), enabling physicians to prescribe health apps. Statutory health insurance companies will reimburse the costs for health apps to the individuals they insure, which is an internationally unique system of health app usage encouragement [6].

First, to research users’ estimated willingness to accept a certain monetary compensation payment—a digital dividend—for sharing self-tracked health data and second, to determine the main factors for such a trade, we conducted a discrete choice experiment (DCE) in Germany. DCE is a choice-based survey method used to research people’s preferences for different options in realistic choice scenarios, contrary to asking them directly about their preferences. We chose this method because people tend to have strong opinions about selling personalized data when asked directly [3]. Previous experiments in lab settings led to demands for high monetary compensation for personal data [7–9]. We used a specific type of DCE, separated adaptive dual response (SADR), to get a more realistic picture of preferences. The experiment was launched before the outbreak of COVID-19 in Germany.

Therefore, we are going to answer the research question under which circumstances wearable and health app users would accept a digital dividend for their self-tracked health data. To answer the research question, we conducted two identical studies with different prices to test the general robustness of price estimates, as we expected people to overestimate the monetary value of their self-tracked data.

## Background

Research about price expectations for self-tracked health data is still sparse. As the RKI COVID-19 data donation app was found to fight the severe social and physical effects of the pandemic, the data donation model enjoyed a significant trust advantage. Hence, there is already severe criticism of the RKI data donation model because the donated data is not pseudonymized directly. This leaves a security loophole and facilitates data misuse as well as identification of personalized fitness histories [10]. As soon as the RKI loses people’s trust in its approach to data processing and the threat of the COVID-19 pandemic has eased, the donation model might become obsolete. Even though Skatova and Goulding argue that people donate their data mainly for altruistic reasons [11], the donation model does not reach a large-scale and diverse group of people [10], which would be needed for big data research with self-tracked data.

New technologies such as artificial intelligence and big data offer unprecedented possibilities for use of self-tracked health data in research and development (R&D), which would therefore decrease the costs and duration of expensive control group studies. This might be the next milestone in medical research. Self-tracked data is going to provide new insights using prescriptive, descriptive, predictive analysis and simulations [12–14], potentially improving treatment quality, preventive care, and diagnostics [13]. Hence, research about incentive systems to trigger a large and diverse group of users to share their data for R&D purposes is essential. Bataineh et al. criticized the market for personal data because it shows a significant imbalance [15]. App users are currently not compensated for the provision of their data, and the authors further criticized the lack of an adequate platform to monetize and trade self-tracked data [15]. The question about the compensation that people are willing to accept remains unsolved. This is despite the fact that there have already been some research attempts at answering this question, as described in the following section. However, an SADR study, i.e. a well-aligned, trade-off-based approach, has not yet been performed, even though the settings in this type of study allow for more realistic results.

Wathieu and Friedman analyzed the lack of confidence in online services and app providers and the general rejection of personal data trade [16]. Spiekermann et al. researched the conditions of the international market for personal data. They concluded that people tend to overestimate the monetary value of their data because they do not feel comfortable with the possibility of their data being linked back to them [17]. Many people have little knowledge about the market for personal data and barely know that the data of a single person is rather worthless. However, statistical clustering and digital phenotyping might help to find patterns or gain new insights about the occurrence of different chronic diseases [17]. Therefore, it might be difficult to find a realistic price for health data, depending on personal attitudes and preferences. People often claim strong data privacy concerns, but over 30 million Germans use the payback system, a commercial bonus program offered by the American Express Group. This program enables the trade of consumer shopping information for bonus points. One bonus point equals one Euro Cent [18].

Cvrcek et al., Grossklags and Acquisti, and Hubermann et al. conducted experimental studies to estimate willingness to accept (WTA) and willingness to pay (WTP) for the disclosure of different personal information [9–11]. WTA demonstrates the minimum amount of money one has to offer an individual in order for them to give up a specific good or service. At the same time, WTP illustrates the maximum amount of money an individual would be willing to pay for a service or a good [19]. Many studies showed a significant disparity between WTA and WTP for the same good, which may relate to some form of loss aversion [19]. Aquisti et al. state that “what people say their data is worth depends critically on the context in which they are asked” [20]. This means that people’s perception of their data’s monetary value depends to a large extent on whether they are asked for their data to be protected or whether they are asked to sell their data.

Estimating WTA for PGHD seems difficult because personal price expectations are influenced by opinions, experience, settings, and attitudes. The amount may differ for each individual and depends on how emotionally charged the information is. For example, Cvrcek et al. discovered that participants would agree to publish their smartphone location points for 43.00€ [7]. Grossklags & Acquisti, on the other hand, conducted a quiz with participants and found that people would publish their quiz results on average for $7.06 USD and their personal information for $31.80 USD [8]. The WTP for protecting the same data was below one USD. When asked about number of sexual partners, the WTA averaged $2291.30 USD, and the WTP to protect the same data was $12.10 USD [8]. Such examples illustrate the significant gap between people’s WTP and WTA for data privacy, which results from missing transparency about data processing and a lack of data privacy education. Many app providers offer their services free of charge for the user but sell user data to third parties or use advertisements on their platforms to finance their businesses. Users are often unconscious of these financing models. Furthermore, WTA depends to a large extent on the type of information provided. Hubermann et al. asked participants to auction information about their age, weight, and height for their study. They concluded that participants with a higher body mass index demanded higher prices for the information about their weight than people with a body mass index below average [9].

These experimental studies are groundbreaking in drawing a bigger picture about the practicability of a digital dividend. Participants were not asked to share their continuous real world health data in other experimental studies. In some studies, researchers were able to link the information back to the individuals [7–9,19], whereas in reality, data should be stored and sold anonymized or at least pseudonymized. Making personalized data publicly available often leads to a feeling of embarrassment and, therefore, leads to people demanding higher prices for their information [20]. In most experiments [8,9,19], participants would agree to a one-time data trade for a single payment, whereas a continuous data exchange would be more feasible for large research projects; this is why a continuous digital dividend seems more realistic than a single reward for our study. The literature shows that transparency about data selling to third parties, as well as the nature of health data in particular, seem to be essential factors for the dividend demanded [3,9,16,21]. Research about different incentive systems to trigger people to share their data with different R&D institutions is becoming increasingly important because large-scale self-tracked health data simulations could become a game changer in the fight against the COVID-19 pandemic and beyond. This paper contributes to the current political and academic debate about a digital dividend for self-tracked health data, offering a different and more realistic methodological setting.

## Data and methods

To research the digital dividend demanded for self-tracked data, we conducted a DCE. Contingent valuation was no option for the purpose of our study because users had no price demand experience. Our study settings did not allow respondents to directly state their WTA but rather asked about their preferences for different real-world scenarios [22]. If asked directly, people with strong data privacy concerns and opinions tend to block any imagination about selling their self-tracked health data in surveys or interviews, leading to observations of extreme response behavior. Previous experiments in lab settings led to high demands for monetary compensation [8]. Another method might have been a quantitative data analysis with proxy values for data prices. Databases for digital data prices barely exist. Known models to estimate the price of personal data might include payback estimates [18]. However, these are not suitable to calculate the price of health data because health and health data are special goods. We expected that patient generated health data would be more valuable for the stakeholders than shopping data. In our online experimental survey, we showed respondents various offers for their self-tracked data. They had to decide which combination of circumstances they preferred and whether they accepted the offer. Based on these decisions, we estimated the extent to which each attribute level contributed to the observed decision [23].

Between May and October 2019, we conducted two pre-studies with 35 and 100 respondents prior to the final data acquisition, targeting primarily students from our University’s network, who most likely used health apps or wearables to track health. Hence, we ran a power analysis using the results from the 100-respondent pre-test according to Bekker-Grob et al. [24]. The results showed that we needed a minimum of 192 respondents for each questionnaire when applying a significance level of p ≤ 0.05. In December 2019, we conducted the main study over a period of three weeks with 842 respondents, and a second, identical study with 272 respondents which contained lower prices. We launched the second study with lower prices to test the price estimates of the main study for robustness. We carried out the two studies through a professional panel provider, Norstat GmbH, and targeted German speaking respondents aged 18 years to 99 years. By signing their terms and conditions, we committed ourselves to follow their code of ethics and the ESOMAR guidelines. In line with our power analysis, more than 192 respondents participated in either of the questionnaires.

### Attributes and attribute levels

We conducted a scoping review, which can be found in a previous study [25], to decide on attributes and levels. Additionally, we used purposeful sampling for this study to identify the current state of experimental studies on the monetary value of health data [26]. To further validate the arising attributes and levels, we run an online survey about the importance of different attributes. Sixteen individuals participated in this survey; most of them were between 26 and 35 years old, users of health apps or wearables, and generally fit. Participants had to rank different attributes according to their perceived importance for the decision to sell their self-tracked health data. *Type of data storage* and *data sales to third* parties were the most important attributes for the decision to sell self-tracked data. The attribute *app provider* was not important to the respondents because the decision to trust an app provider had previously taken place when downloading or purchasing the app. Most participants claimed that the specific *type of data* had a significant effect on the decision. We performed the first pre-study with 35 respondents and consequently eliminated the attribute *type of data storage* because respondents would solely focus on this attribute and ignore all others. Because of current public data protection initiatives, it is highly unlikely that stakeholders in the health care sector would be allowed to store personalized data. The second pre-study with 100 respondents led to the final validation of four attributes: monthly bonus payment (5€–75€ or 10€–31€), stakeholder (health insurer, pharmaceutical, and medical device company or universities), type of data (motion and cardio data, nutrition and lifestyle data or all data with health relevance) and data sales to third parties (raw data is going to be sold for profit, raw data is not going to be sold for profit or raw data is not going to be sold, statistically processed data is going to be sold). S1 Table shows the attributes and levels with which the respondents were confronted in the DCE survey [S1 and S2 Figs].

### Experimental design

The experimental design of the DCE simulates realistic decision scenarios, which illustrate the preferences of the respondents. Given that sharing health data is emotionally charged, we used the DCE alternative SADR to reduce extreme response behavior. The SADR method requires respondents to choose first among forced choice options and second among free choice options. Using the respondent’s choice probability, which depends on the utility of the scenario selected and the other competing scenarios within the choice set, we estimated the minimum price at which respondents would sell their self-tracked data [27].

DCEs have been observed as being “popular preference elicitation methods, yet they can suffer from context effects, extreme response behavior, and problems with estimating consumers’ willingness to pay” [28]. SADR outperforms traditional DCEs because the method first measures the attractiveness of all attributes and uses this information to adaptively identify towards which offer a respondent becomes indifferent when accepting it [28]. Thus, SADR provides advantages in situations in which the threshold between accepting and not accepting an offer is heterogeneous across respondents. We conducted the study using the Dynamic Itelligent Survey Engine (DISE) online platform [29].

We first presented ten choice sets to the respondents, for which we forced them to decide between three different offers. We used a D-efficient linear probability model to estimate the main effects of attribute levels. The design D-efficiency is 97%. The reason why we chose to include an extraordinary amount of monthly bonus payment levels is because of price uncertainties. Estimating a realistic price for personal health data is difficult because there is no market price yet, so we chose eight levels for this attribute, given the unaffected design efficiency. We assumed a linear relationship for the price. The higher the offered price, the more likely data will be sold. The attributes are independent, and a level balance is given [30].

In the second part of the experiment, we adaptively generated six offers and asked respondents, whether they would accept or not. Schlereth and Skiera stated that the adaptive offer generation mechanism ensures that decisions are not biased through endogeneity [28]. To avoid order effects, we randomized choice set order across respondents [31,32].

To test the robustness of the price estimates obtained from the main study, a second study was run in parallel with an alternative range of possible prices to account for the anchoring effect. Subjects were randomly assigned to either the main study or to the second study. Bevan & Pritchard described the phenomenon as follows: “Anchor effects are systematic changes in the judgement of series stimuli” [33]. This means that people who have no price expectations tend to take the given prices as an orientation point or an anchor. Hence, this means that the price expectations could be strongly correlated with the prices offered during the experiment.

### Data

In December 2019, 842 respondents participated in the main study, and 272 respondents participated in the second study of our online experiment [S1–S3 Raw Data] through the online panel Norstat GmbH. Norstat GmbH confirmed that our survey followed the ESOMAR international code on marketing, opinion, social research, and data analytics. All respondents’ data was stored pseudonymized on our University server in Germany. We requested no personal information from the respondents, and the answer to socio-demographic questions was voluntary and could be left out. Participation in the survey was also voluntary. Minors (<18 years old) did not participate. Respondents agreed to the terms and conditions before participation, first with Norstat GmbH and then when participating in our survey. We ensured the quality of answers by integrating an attention question, which resulted in the exclusion of respondents who did not attentively read the questionnaire. The attention question required that option B be chosen out of options A, B, C, and D. The main study contained a price range from 5€ to 75€ for certain circumstances to share self-tracked health data with different stakeholders. The sample reflected a heterogeneous group of German citizens [S5 Table].

Our sample consists of 47% (397/842) female respondents and therefore shows a gender balance. About 34% (298/842) of the respondents use health apps or wearables to track their health, and just about 4% (36/842) share their data with family, friends, physicians, or on social media platforms. Still, 73% (611/842) of the respondents would share their self-tracked data with physicians for better diagnostics and therapy. To ascertain the level of trust in different agents with their personal health data, respondents were instructed to distribute 100 points so that the agents trusted most would score the highest number of points. The results showed that physicians scored on average 57.2 trust points, health insurance companies 15.5, universities 14.7, medical device companies 4.1, government institutions 3.5, pharmaceutical companies 3.3 and social media companies 1.7. This means that main research institutions in particular, like the pharmaceutical industry, government institutions, and medical device companies, score very low trust levels within German society.

### Econometric modeling

We estimated choice probabilities using methods similarly to those used by Schlereth et al. [32]. We ran a random utility framework to evaluate individual choice preferences [34]. Hence, we estimated a hierarchical Bayes multinomial logistic regression model using the software MATLAB [31]. Using an iterative process, the model evaluates the results on two levels; aggregate and specific behavior [31]. Significance levels and t-statistics are usually not assessed because through a large number of iterations, the ex post distribution is almost always significant [31]. The estimation assumes that a respondent *h* desires offer *i*, which maximizes his or her utility, given the utility function:

$$u_{h,i}=v_{h,i}+\varepsilon_{h,i}$$ (1)

[32]

*u*_*h*,*i*_ is the utility of a respondent to choose a digital dividend offer under specific circumstances for his or her self-tracked health data. *v*_*h*,*i*_ is the deterministic part of the utility and contains all the observable information shown in the choice-sets, like all levels of the different attributes, while *ε*_*h*,*i*_ is the error term and contains all unobserved information [32].

To determine the log-normal probability distribution of individuals selling their data under specific conditions to one stakeholder versus another, we use the following formula:

$${Pr}_{h,a}(i)={Pr}_{h,a}(i|1st\;choice)∙\;{Pr}_{h}(i|2nd\;choice)=\frac{\text{exp}(v_{h,i^{\prime }})}{\sum_{i^{\prime }\in C_{a}}\text{exp}(v_{h,0})}∙\frac{\text{exp}(v_{h,i})}{\text{exp}(v_{h,0})+\text{exp}(v_{h,i})}$$ (2)

(*h* ∈ *H*, *a* ∈ *A*) [32]

*Pr*_*h*,*a*_(*i*) is the probability the participant *h* chooses the pricing plan *i* for each choice-set *a* [32]. *H* is the set of all respondents, *A* refers to all choice-sets in the experiment and *C*_*a*_ to the decision alternatives for prices shown in the first question of the choice-set [32]. Formula 2 illustrates a non-sequential model, hence we assume that two choices in a choice-set are independent by multiplying both corresponding probabilities [32,35]. The multinomial logit model is conducted to resolve for the differences in consistency between the forced-preference question set and the free-acceptance question set [36], using the following formula:

$$L_{h}=\prod_{a\epsilon A}\prod_{i\epsilon C_{a}}{\left(\frac{\text{exp}\left(v_{h,i}\right)}{\sum_{i^{\sim }\in C_{a}}\text{exp}\left(v_{h,i^{\sim }}\right)}\right)}^{d_{h,i,a}}∙\;\prod_{i^{\prime }\epsilon C_{h^{\prime }}}\left({\left(\frac{\text{exp}\left(v_{h,i^{\prime }}\right)}{\text{exp}(v_{h,0})+\text{exp}\left(v_{h,i^{\prime }}\right)}\right)}^{d_{h,i^{\prime }}}∙{\left(\frac{\text{exp}\left(v_{h,0}\right)}{\text{exp}(v_{h,0})+\text{exp}\left(v_{h,i^{\prime }}\right)}\right)}^{{1-d}_{h,i^{\prime }}}\right)$$ (3)

(*h* ∈ *H*) [32]

*L*_*h*_ is the likelihood for predicting respondent *h*’s observed choices [32]. The first part computes the probability that the respondent observes the decisions *d*_*h*,*i*,*a*_. The second part estimates the probability of accepting (*d*_*h*,*i*′_ = 1) and not accepting (*d*_*h*,*i*′_ = 0) a concrete offer *i* from his or her set of individually seen offers *C*_*h*′_ [32].

In a next step, we calculated the estimated *WTA*_*h*,*i*_ of respondent *h* for offer *i* as a minimum marginal price people would accept to share their self-tracked health data, using the following formula:

$${WTA}_{h,i}=\frac{X_{i}∙\beta_{h}}{\omega_{h}}$$ (4)

[27]

In this formula, *X*_*i*_ is the design vector, *β*_*h*_ is the preference vector, and *ω*_*h*_ is the price parameter [27]. This way, we measured whenever a respondent became indifferent between accepting a monthly bonus payment offer or rejecting it, which implies that the utility of selling self-tracked data at a specific price, equal to his or her WTA, has the same price as the utility of not selling [27].

## Results

### Parameter estimates

We estimated the parameters using a hierarchical Bayes multinomial logistic model, using the software MATLAB [32,35]. The ß-parameter depicted in S2 Table measured the relative importance of a certain level of an attribute over another for each respondent’s choice [27]. Through a careful selection of attributes and levels prior to the study, we offered as realistic scenarios as possible from which respondents could choose [37].

Data sales to third parties scored by far the highest importance weight (44.17%). If raw data is going to be sold to third parties, it is highly unlikely that individuals will choose to share their data with different agents. Hence, the sharing of personalized data is a value-laden topic. The results showed that people were sensitive to transparency about data processing and data security because they are afraid of discrimination. This supports the claims of Wathieu and Friedman that people are very concerned about indirect data security due to missing transparency during data processing [16]. The second most important attribute was the stakeholder. The pharmaceutical industry, medical device companies, and health insurers seem to suffer from large trust deficits. However, monthly bonus payments had an importance weight of 20.70%.

The results showed that a digital dividend could be an effective economic incentive system to motivate people to share their data for R&D purposes with different agents. The type of data was the least important attribute. People were less willing to share nutrition and lifestyle data than just motion and cardio data. We could not find any indicators that socio-demographic factors, such as age or gender [S6 Table], influenced the results in any particular way [38].

### Willingness to accept

S3 Table shows the estimated WTA participants demanded from different stakeholders if data was not sold to third parties and all their data with health relevance was shared in our experiment.

The price expectations seem high but show that a digital dividend could be a working economic incentive system as many respondents were ready to share their data for a certain price. When considering a scenario in which all data is going to be shared and raw data is not further sold to third parties, universities would have to pay 145.66€/month to use the data. Health insurers would have incremental costs of 31.75€/month and pharmaceutical, and medical device companies would have incremental costs of 92.15€/month compared to universities. These price differences might be explained by a trust delta between the different stakeholders.

When plotting the estimated WTA of the scenario in which all data is shared and no data is sold to third parties with different stakeholders in histograms [S3–S5 Figs], we observed a bimodal or a u-shaped distribution between low price expectations (0€–5€) and high price expectations (>50€). When the scenario is offered by the health insurers, 28.62% (241/842) demanded 0€–5€ and 48.81% (411/842) demanded >50€. Similar distributions can be observed for the pharmaceutical and medical device companies as 38.95% (328/842) demanded 0€–5€, and 48.81% (344/842) demanded >50€, and the universities, as 45.97% (387/842) demanded 0€–5€ and 32.67% (275/842) demanded >50€. These differences between the two extreme poles of nearly donating self-tracked data and demanding high prices explain the high average estimated WTA and demonstrate that people have no consolidated price expectations or general knowledge about the monetary value of patient generated health data.

A more realistic price scenario might be 15€–20€ per month, which was tested by Facebook in the “Atlas” experiment [39]. However, the project was criticized because teen users were asked to download a VPN, which tracked and stored personalized data from all other apps, including messaging and social media, on the device [39]. Facebook paid the participants $20/month USD.

In our study, considering a scenario if all data with health relevance was sold and no data sales to third parties took place for 20€/month; 54.91% of the participants would accept the offer from the university, 46.65% from the health insurer, and 38.87% would accept the offer from pharmaceutical or medical device companies. When offering 15€/month to share just cardio and fitness data and no data sales to third parties, 60.60% would accept the offer from the university, 52.45% from the health insurer, and 45.02% from the pharmaceutical or medical device company. We observed that people generally have high price expectation for their digital dividend because they overestimate the monetary value of their self-tracked data.

### Pricing robustness test

To test the estimated WTA of the main study for robustness, we assigned respondents randomly to one of two identical questionnaires with varying price ranges to account for the anchoring effect. From among a total of 1,114 participants, 842 were directed to the main study with a price range of 5€–75€ and 272 were directed to the second survey with a reduced-price range of 10€ –31€ (S4 Table).

Comparing the results of the main study with the second study, we observed an anchor effect as we got lower WTA estimates in the study with lower initial prices. The results of the second study showed that people did not have any experience with or expectation of the monetary value of their health data and yet overestimated their price. Furthermore, our output validated the argument that most stakeholders in the health care sector suffer from a lack of trust, which is why health insurers and pharmaceutical and medical device companies would need to pay higher prices for patient generated health data than universities.

In a scenario in which all data with health relevance was shared for 20€/month, and no data sales to third parties took place, 62.86% accepted the offer from the universities, 56.06% from the health insurers, and 45.51% from the pharmaceutical and medical device companies. When offering 15€/month for sharing just cardio and fitness data and no data sales to third parties took place; 63.94% accepted the offer from the universities, 57.65% from the health insurers, and 47.28% from the pharmaceutical and medical device companies. These results were very similar to the ones from the first study and showed the general robustness of the data.

The robustness test showed that the design of the offer and the amount of the digital dividend demonstrated to participants had an observable effect on the respondents’ price expectations. Even though the prices might be high, we observed that a monetary incentive could be an accepted instrument to continuously acquire a large and diverse amount of self-tracked health data.

## Discussion

In this research, we complemented the current political discussion about a potential legal setting for digital dividends. The results of our analysis clearly showed that participants would share their health data for a digital dividend if no data sales to third parties occurred and stakeholders were transparent about data storage and processing. Hence, we derived from our results that German stakeholders in the health care sector in general and pharmaceutical companies in particular suffer from a significant lack of trust, which directly influences the price expectations for data. We agree with Spiekermann et al. and Wathieu and Friedman that people have difficulty estimating the monetary value of their data because they have little or no knowledge of its statistical use in R&D [16,17]. However, we used a different experimental setting than previous studies have because we offered a monthly bonus payment to the participants for a continuous data exchange, rather than single payment for a one-time disclosure of personal data.

Our analysis also showed that socio-economic factors are either irrelevant to or play no role in whether a person would share his or her data with a health care stakeholder, if the stakeholder earns enough trust and is transparent about data use and processing. This conclusion might be especially interesting for the enhancement of pharmaceutical research because self-tracked data from a diverse group of people might complement or replace control group studies [40–42].

The robustness test showed an anchor effect for the prices [33], i.e. that people had no expectations or knowledge about the price of digital health data. Hence, this might be a chance to introduce a digital dividend because the market may set the price at first. The estimated WTA levels resulting from the study were higher than the attribute levels, which again signals the uncertainty about prices and the participants’ diverging expectations. Future research should address the stakeholder side because we do not know if the participants’ estimated WTA matches stakeholders’ WTP. Hence, given the COVID-19 crisis and a conceivable resulting change of long-run preferences, a second study with lower prices should be repeated in the future. Price expectations might have changed because of the sensitization for the need to enhance and complement research with big data PGHD studies after the COVID-19 crisis.

## Summary and conclusion

In summary, stakeholders within the health care sector should engage in more transparency about data storage and data sales to third parties. Such an effort can encourage people to share their self-tracked data with them for R&D. These companies seem to enjoy a low level of trust concerning the processing of personal data. People tend to overestimate the monetary value of their health data, which resulted in high prices demanded. Respondents were generally sensitive to further data sales to unknown third parties.

Nevertheless, the results showed that a digital dividend could be an accepted instrument to convince people to share their data for R&D purposes, having an importance weight of nearly 21%. The pharmaceutical industry in particular would benefit from the inclusion of big data PGHD research. There are high incremental costs for health insurance, pharmaceutical, and medical device companies in comparison to universities. Under certain conditions, 54.91% of the participants would sell their self-tracked data to universities, 46.65% to health insurers, and 38.87% to pharmaceutical or medical devices companies for 20€/month. When considering the scenario that all data is sold and raw data was not further sold to third parties, then universities would have to pay 145.66€/month to use the data. Health insurers would have incremental costs of 31.75€/month and pharmaceutical and medical device companies would have incremental costs of 92.15€/month compared to universities. For agents who would like to buy self-tracked data, it would be advisable to encourage more transparency and educational campaigns about their data processing and data security strategies to increase people’s trust by dissolving the fear of personal discrimination.

A continuing success of the data donation model in Germany is questionable because people were triggered by the events of the COVID-19 crisis when engaging with the RKI data donation app. The discussions about the data security of the RKI data donation app already sparked fear about a lack of transparency and possible discrimination through data misuse. Transparency, trust, and monetary compensations are effective long-term measures to convince a diverse and large group of people to share their high-quality data. Hence, this is also an encouraging result as PGHD-research may help identify the reasons for chronic diseases or severe conditions. The public discussion about patient generated health data usage in R&D might also increase general health app usage and, therefore, health education through self-monitoring.

## Supporting information

S1 Fig Forced choice question design. (DOCX)

S2 Fig Free choice question design. (DOCX)

S3 Fig WTA from health insurers for all data and no data sales to third parties. (DOCX)

S4 Fig WTA from pharmaceutical and medical device companies for all data and no data sales to third parties. (DOCX)

S5 Fig WTA from universities for all data and no data sales to third parties. (DOCX)

S1 Table Attributes and levels. (DOCX)

S2 Table Parameter estimates and importance weight of attributes—Main study. (DOCX)

S3 Table Willingness to accept to share self-tracked health data—Main study. (DOCX)

S4 Table Willingness to accept to share self-tracked health data—Second study. (DOCX)

S5 Table Descriptive statistics. (DOCX)

S6 Table Multiple linear regression models. (DOCX)

S1 Raw Data DISE output. (XLSX)

S2 Raw Data ß-parameter. (XLSX)

S3 Raw Data XLM file survey. (XML)
