# Supplementary material for: Pricing through health apps generated data—Digital dividend as a game changer: Discrete choice experiment
Source: PLoS One. 2021 Jul 26;16(7):e0254786. doi: 10.1371/journal.pone.0254786 (PMC8312968; doi:10.1371/journal.pone.0254786)
Supplement: S2 Fig — (DOCX) [file pone.0254786.s002.docx]

**S8 Figure. Free Choice Question Design**


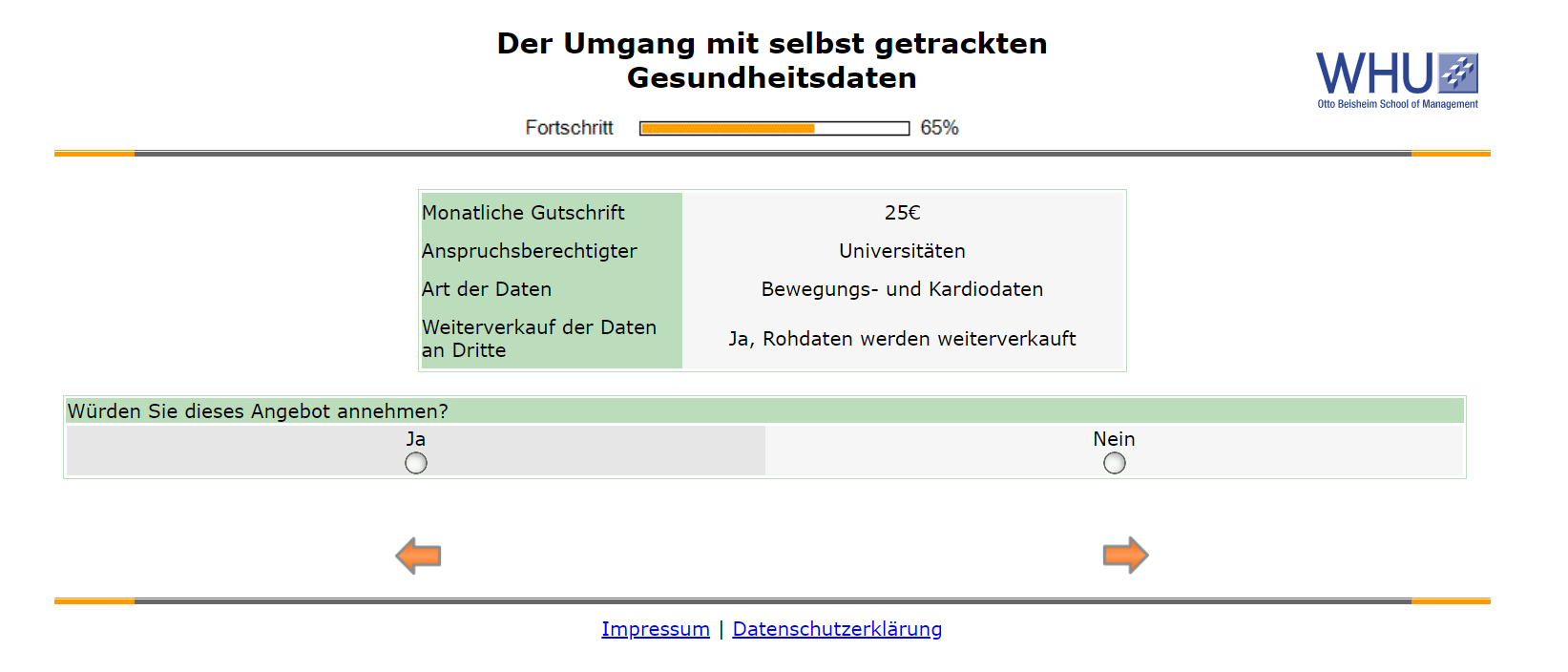
Source: Own Depiction Survey on Online Survey Engine DISE ( <https://www.dise-online.net/surveystart.aspx?config=HAPWsurvey>)
