# Supplementary material for: Pricing through health apps generated data—Digital dividend as a game changer: Discrete choice experiment
Source: PLoS One. 2021 Jul 26;16(7):e0254786. doi: 10.1371/journal.pone.0254786 (PMC8312968; doi:10.1371/journal.pone.0254786)
Supplement: S4 Fig — (DOCX) [file pone.0254786.s004.docx]

**S10 Figure. WTA from Pharmaceutical and Medical Device Companies for All Data and no Data Sales to Third Parties**


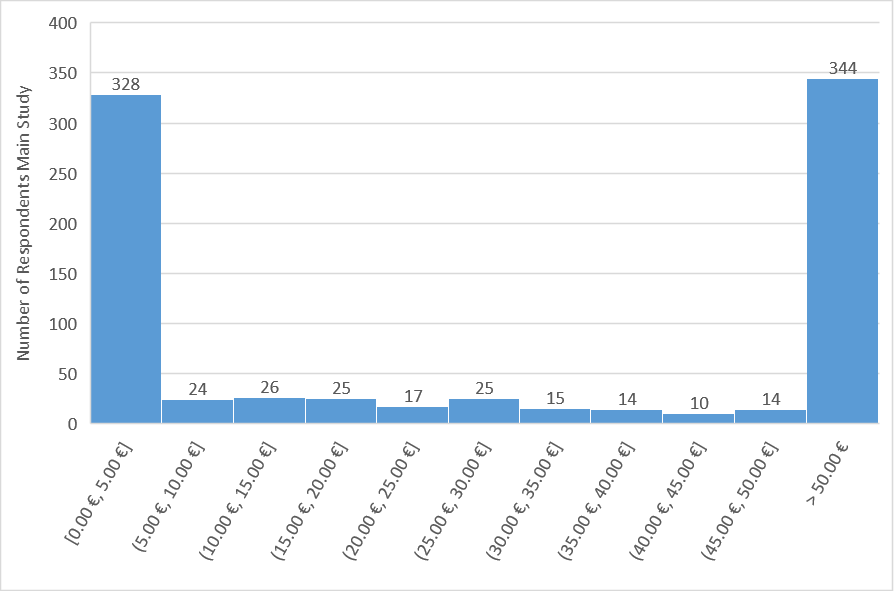


Source: Own Depiction
