# Supplementary material for: Pricing through health apps generated data—Digital dividend as a game changer: Discrete choice experiment
Source: PLoS One. 2021 Jul 26;16(7):e0254786. doi: 10.1371/journal.pone.0254786 (PMC8312968; doi:10.1371/journal.pone.0254786)
Supplement: S1 Table — (DOCX) [file pone.0254786.s006.docx]

**S1 Table. Attributes and Levels**

|  | Attributes | Levels | | | | | | | |
| --- | --- | --- | --- | --- | --- | --- | --- | --- | --- |
| Main Study | Monthly Bonus Payment | 5€ | 15€ | 25€ | 35€ | 45€ | 55€ | 65€ | 75€ |
| Second Study | Monthly Bonus Payment | 10€ | 13€ | 16€ | 19€ | 22€ | 25€ | 28€ | 31€ |
|  | Stakeholder | Health Insurer | Pharmaceutical and Medical Device Companies | Universities |  |  |  |  |  |
|  | Type of Data | Motion and Cardio Data | Nutrition and Lifestyle Data | All Data with Health Relevance |  |  |  |  |  |
|  | Data Sales to Third Parties | Yes, raw Data is going to be sold for Profit | No, raw Data is not going to be sold for Profit | Raw Data is not going to be sold, statistically processed Data is going to be sold |  |  |  |  |  |

Source: Own Depiction.
