# Supplementary material for: Pricing through health apps generated data—Digital dividend as a game changer: Discrete choice experiment
Source: PLoS One. 2021 Jul 26;16(7):e0254786. doi: 10.1371/journal.pone.0254786 (PMC8312968; doi:10.1371/journal.pone.0254786)
Supplement: S2 Table — (DOCX) [file pone.0254786.s007.docx]

**S2 Table. Parameter Estimates and Importance Weight of Attributes - Main Study**

| **Attribute** | **Level** | **ß-Parameter Average (Standard Deviation)** | **Importance Weight (Standard Error)** |
| --- | --- | --- | --- |
| **Constant Parameter** |  | -2.45 (2.37) |  |
| **Monthly Bonus Payment** | 5 EUR - 75 EUR | 0.09 (0.42) | 20.70% (1%) |
| **Stakeholder** | Health Insurer | -0.06 (1.03) | 23.82% (1%) |
|  | Pharmaceutical and Medical Device Companies | -0.55 (0.73) |  |
|  | Universities | 0.61 (0.76) |  |
| **Type of Data** | Motion and Cardio Data | 0.23 (0.45) | 11.31% (0%) |
|  | Nutrition and Lifestyle Data | 0.12 (0.25) |  |
|  | All Data with Health Relevance | -0.35 (0.50) |  |
| **Data Sales to Third Parties** | Yes, raw Data is going to be sold for Profit | -2.56 (1.91) | 44.17% (1%) |
|  | No, raw Data is not going to be sold for Profit | 1.54 (1.14) |  |
|  | Raw Data is not going to be sold, statistically processed Data is going to be sold | 1.03 (0.92) |  |

Source: Own Depiction
