# Supplementary material for: Pricing through health apps generated data—Digital dividend as a game changer: Discrete choice experiment
Source: PLoS One. 2021 Jul 26;16(7):e0254786. doi: 10.1371/journal.pone.0254786 (PMC8312968; doi:10.1371/journal.pone.0254786)
Supplement: S3 Table — (DOCX) [file pone.0254786.s008.docx]

**S3 Table. Willingness to Accept to share Self-Tracked Health Data – Main Study**

| **Stakeholder** | **Scenario** | **Mean Willingness to Accept (EUR/Month), Price Range 5€ -75€(Standard Error)** |
| --- | --- | --- |
| Health Insurer | - All Data with Health Relevance - Raw Data is not going to be sold for Profit | 177.41 EUR  (10.71 EUR) |
| Pharmaceutical and Medical Device Companies | - All Data with Health Relevance - Raw Data is not going to be sold for Profit | 237.80 EUR  (14.36 EUR) |
| Universities | - All Data with Health Relevance - Raw Data is not going to be sold for Profit | 145.66 EUR  (10.69 EUR) |

Source: Own Depiction
