# Supplementary material for: Pricing through health apps generated data—Digital dividend as a game changer: Discrete choice experiment
Source: PLoS One. 2021 Jul 26;16(7):e0254786. doi: 10.1371/journal.pone.0254786 (PMC8312968; doi:10.1371/journal.pone.0254786)
Supplement: S5 Table — (DOCX) [file pone.0254786.s010.docx]

**S5 Table. Descriptive Statistics**

| **Independent Variables** | | **Distribution within Sample/Mean** | | **German Average (destatis.de)** |
| --- | --- | --- | --- | --- |
| Gender | Female | | 53 % | 51 % |
|  | Male | | 47 % | 49 % |
|  | Divers | | 0.1 % |  |
| Age | 15-25 years | | 8 % | Less than 20 years: 18.4% |
|  | 26-35 years | | 12 % | 20-40 years: 24.6% |
|  | 36-45 years | | 17 % | 40-60 years: 28.8 % |
|  | 46-55 years | | 22 % | 60-80 years: 21.7 % |
|  | 56-65 years | | 18 % | 80 and older: 6.5% |
|  | older than 65 years | | 23 % |  |
| Monthly Household Net Income | N/A | | 11 % |  |
|  | Up to 500 EUR | | 3 % | Less than 1300 EUR: 16.3 % |
|  | 500 - 999 EUR | | 8 % | 1300 EUR - 1700 EUR: 9.1 % |
|  | 1000 - 1999 EUR | | 25 % | 1700 EUR - 2600 EUR: 20.6 % |
|  | 2000 - 2999 EUR | | 21 % | 2600 EUR - 3600 EUR: 17.8 % |
|  | 3000 - 3999 EUR | | 18 % | 3600 EUR - 5000 EUR: 17.5 % |
|  | 4000 - 4999 EUR | | 9 % | 5000 EUR - 18000 EUR: 18.6 % |
|  | more than 5000 EUR | | 5 % |  |
| Education | No High School Diploma | | 0.4 % | 4 % |
|  | Lower Secondary Education | | 12 % | 30.4% |
|  | Secondary Education | | 38 % | 23.1% |
|  | Upper Secondary Education | | 9 % | Fachhochschul- oder |
|  | General Qualification for University Education | | 18 % | Hochschulreife: 31.9 % |
|  | University Degree (BA, MA or higher) | | 23 % | 17.7 % |
| Source: Own Depiction | | | | |
