# Supplementary material for: Pricing through health apps generated data—Digital dividend as a game changer: Discrete choice experiment
Source: PLoS One. 2021 Jul 26;16(7):e0254786. doi: 10.1371/journal.pone.0254786 (PMC8312968; doi:10.1371/journal.pone.0254786)
Supplement: S6 Table — (DOCX) [file pone.0254786.s011.docx]

**S6 Table. Multiple Linear Regression Models**

| Coefficients | Dependent Variable: ß-parameter ‘Data Sales to Third Parties’ | Dependent Variable: ß-parameter ‘Health Insurer’ | Dependent Variable: Importance Weight ‘Price’ |
| --- | --- | --- | --- |
| Constant | 0.005 **  (0.631) | 0.509  (0.340) | 0.0269 *  (0.078) |
| Sex/Male | 0.195  (0.134) | 0.532  (0.072) | **0.005 ****  **(0.017)** |
| Sex/Divers | **0.085 .**  **(1.906)** | 0.101  (1.028) | 0.466  (0.236) |
| Age | **0.039 ***  **(0.045)** | 0.943  (0.024) | 0.293  (0.006) |
| Health App & Wearable Usage/ No | 0.308  (0.147) | **0.002 ****  **(0.079 )** | **0.002 ****  **(0.018)** |
| Fitness | **0.061 .**  **(0.040)** | 0.181  (0.022) | 0.519  (0.005) |
| Health | 0.170  (0.041) | 0.903  (0.022) | 0.376  (0.005) |
| Monthly Household Net Income | 0.691  (0.037) | **0.031 ***  **(0.020)** | 0.625  (0.005) |
| Education | **0.035 ***  **(0.048)** | 0.195  (0.026) | 0.895  (0.006) |
| Perceived DSGVO Importance | **0.006 ****  **(0.046)** | 0.100  (0.025) | 0.108  (0.006) |
| BMI | 0.987  (0.013) | 0.656  (0.007) | 0.108  (0.002) |
| Sharing of Fitness or Health Data/ No | 0.231  (0.333) | 0.578  (0.180) | 0.697  (0.041) |
| R-squared  Adjusted R-squared  F-statistic | 0.036  0.023  2.806 on 11 and 830 DF | 0.031  0.019  2.439 on 11 and 830 DF | 0.039  0.026  3.061 on 11 and 830 DF |

Source: Own Depiction
